# A Rare Subtype of Non-small Cell Lung Cancer: Report of 159 Resected Pathological Stage I–IIIA Pulmonary Lymphoepithelioma-Like Carcinoma Cases

**DOI:** 10.3389/fsurg.2021.757085

**Published:** 2021-10-27

**Authors:** Rong-Rong Jiang, Xiao-Li Feng, Wen-Ting Zhu, Man-Xia Guo, Xue-Li Tan, Xiao-Juan Jiang, Xiao-Meng Dou, Li Liu

**Affiliations:** ^1^Department of Thoracic Surgery, Sun Yat-sen University Cancer Center, Guangzhou, China; ^2^State Key Laboratory of Oncology in South China, Collaborative Innovation Center for Cancer Medicine, Sun Yat-sen University Cancer Center, Guangzhou, China

**Keywords:** lymphoepithelioma-like carcinoma, clinical characteristics, survival, prognosis, stage I–IIIA

## Abstract

**Background:** The current study analyzed resected stage I–IIIA pulmonary lymphoepithelioma-like carcinoma (LELC) cases to define the clinical characteristics, prognosis and long-term outcomes of resected LELC, with the purpose of guiding clinical management for this rare tumor.

**Methods:** Resected stage I–IIIA LELC, adenocarcinoma (ADC) and squamous cell carcinoma (SCC) cases from our center were enrolled. Propensity score matching (PSM) was applied to minimize the selection bias. Overall survival (OS) and disease-free survival (DFS) were compared between groups. Multivariate analyses were performed to identify the prognostic factors, and a nomogram was developed.

**Results:** A total of 159 LELCs, 2,757 ADCs, and 1,331 SCCs were included. LELC, dominated among younger patients and non-smokers. LELC was a poorly differentiated disease that lacked driver gene mutations and was positive for immunohistochemistry indicators of squamous cell lineage. Survival analyses revealed that OS was significantly better for LELC than for other common non-small cell lung cancers (NSCLCs) both before PSM (all *P* < 0.001) and after PSM (all *P* < 0.05). Further analyses revealed that early pathological node stage and preoperative albumin level ≥35 were identified as independent prognostic factors favoring OS and DFS.

**Conclusions:** LELC, dominated among younger and non-smoking populations, lacked driver gene mutations and was positive for immunohistochemistry indicators of squamous cell lineage. The survival outcome of LELC was better than other common NSCLCs.

## Introduction

Primary pulmonary lymphoepithelioma-like carcinoma (LELC), a rare subtype of non-small cell lung cancer (NSCLC), accounts for <1% of all lung neoplasms (1) and was first described in 1987 by Begin (2). According to the World Health Organization (WHO) Classification in 2015, it was removed from the subgroup of large cell lung cancer and reclassified as a unique subgroup of NSCLC (3). Owing to the inherent rarity and the lack of prospective clinical trials, the natural course, prognosis, and management strategy of LELC requires in-depth investigation.

LELC is an Epstein-Barr virus (EBV)-associated and undifferentiated nasopharyngeal-like carcinoma (2, 4, 5). Previous literature demonstrated that most LELC cases were documented in Southeast Asia including Guangdong Province, Taiwan, Hong Kong, and Singapore (6–12). LELC is more prevalent among younger and non-smoking populations without sexual predilection (6, 9, 11, 12). In addition, several clinical series suggested that LELC has a favorable survival outcome when compared with other lung cancers (6, 8, 11, 12). Although many efforts have been devoted to LELC research in the past few decades, the general demographics and prognosis of resected stage I–IIIA LELC remain enigmatic, and larger datasets are warranted to tailor the clinical practice guidelines for this subgroup patients.

In the current study, we retrospectively reviewed 159 resected stage I–IIIA LELC cases to sketch an outline of the clinicopathological characteristics of the disease. The prognostic factors of overall survival (OS) and disease-free survival (DFS) were investigated, and effective predictive nomograms were developed. OS was compared between LELC and other common resected NSCLC both before and after propensity score matching (PSM). We believed that our study may help clinicians estimate individual survival and select a proper treatment strategy.

## Materials and Methods

### Patient Selection

Consecutive resected patients diagnosed with LELC between 1990 and 2016 from the Sun Yat-sen University Cancer Center (SYSUCC) were retrospectively included. Resected patients diagnosed with adenocarcinoma (ADC) and squamous cell carcinoma (SCC) from 2001 to 2016 were also included in this study.

All included cases fit the following criteria: (i) pathologically diagnosed as stage I–IIIA disease and (ii) surgical resection was performed. The exclusion criteria were as follows: (i) previous or concurrent other primary cancers; (ii) age < 18 years old; (iii) underwent neoadjuvant therapy; and (iiii) clinicopathological information was unavailable. In order to exclude LELC from metastasis of undifferentiated nasopharyngeal carcinoma, patients were exposed to nasopharyngoscopy and Epstein-Bar virus-encoded RNA (EBER) test. Additionally, patients with a history of nasopharyngeal carcinoma were also excluded from the study.

This study was conducted in accordance with the Declaration of Helsinki (as revised in 2013). This study was exempted from Institutional Review Board review by the Ethics Committee of SYSUCC as it was a retrospective type and no identifying data were collected. Informed consents of the included patients were also waived by the committee. The authenticity of this article has been validated by uploading the key raw data onto the Research Data Deposit public platform (www.researchdata.org.cn), with the approval RDD number as RDDA2020001729.

### Data Collection

Clinical, pathological and immunohistochemistry (IHC) data were retrieved from patients' medical records. Clinical variables included age, sex, smoking status, tumor history, tumor location, preoperative albumin level, carcinoembryonic antigen (CEA) level, surgical type, and adjuvant therapy. In terms of age, LELC cases were assigned to 2 groups ( ≤60 and >60 years old) based on the optimal cutoff value determined by X-tile software (13). The preoperative albumin and CEA level were dichotomized according to the lower limit of normal. Pathological characteristics included tumor diameter, grade, examined lymph nodes (ELNs), positive lymph nodes (PLNs), T stage, N stage, and TNM stage. ELNs and PLNs were also dichotomized according to the cutoff values determined by X-tile software (13). IHC features included creatine kinase (CK), CK5/6, CK7, thyroid transcription factor (TTF)-1, P63, EBER, epidermal growth factor receptor (EGFR), and anaplastic lymphoma kinase (ALK). EGFR testing was performed by the Amplification Refractory Mutation System (14), and ALK testing was performed by *in situ* hybridization. TNM staging was performed according to the 8th edition of the American Joint Committee on Cancer (AJCC) TNM staging system (15).

### Follow-Up

In general, postoperative follow-up was carried out every 3 months for the first 2 years, every 6 months for the next 3–5 years, and annually thereafter (16). At each follow-up visit, a physical examination and chest and abdominal CT scans were performed (16). If the patient had specific symptoms, the examination was performed as soon as possible for a more careful assessment (16). Follow-up information was updated in October 2020 to determine patients' vital status.

### Adjuvant Therapy

According to the National Comprehensive Cancer Network (NCCN) Guidelines (17), LELC patients who were pathologically diagnosed as Stage II and IIIA diseases were administered with adjuvant chemotherapy. For stage IB patients, only those with high-risk factors (e.g., visceral pleural invasion, poorly differentiated, unknown lymph node status, and wedge resection) received adjuvant chemotherapy. And stage I patients performed regular follow-up strategy but not additional treatments. Due to various reasons (such as stage I disease, economy burden and went to other hospitals for further treatments), patients who were not performed adjuvant therapy in our cancer center were defined as did not perform adjuvant chemotherapy.

### Statistical Analysis

OS was defined as the interval from the date of surgery to the date of death from any cause or the last follow-up. DFS was defined as the time from the date of surgery to the date of tumor recurrence or death from any cause. Categorical variables are presented as number and percentage. Pearson's χ^2^-test or Fisher's exact test was used to compare categorical variables between groups (Fisher's exact test was used when the expected number of events was <5). All survival outcomes were estimated by the Kaplan-Meier method with a log-rank test. A one to one propensity score matching (PSM) method based on age, sex, smoking status, surgical type, ELNs, T stage, N stage, TNM stage, and adjuvant therapy was employed to reduce bias (18), and the caliper was 0.05. Stepwise univariate and multivariate Cox proportional hazards model analyses were used to identify the prognostic factors of OS and DFS. Variables with *P* < 0.05 in the univariate Cox analyses were included in the multivariate Cox analyses. Variables with *P* < 0.05 in the multivariate Cox analyses were included in the nomogram. The concordance index (C-index) was performed to verify the predicted effect of the nomogram (19). R version 3.5.2 (The R Foundation for Statistical Computing, Vienna, Austria; http://www.r-project.org) was involved in developing and validating the nomogram. X-tile software was used to determine the cutoff value (13). IBM SPSS Statistics (version 25.0, IBM Corp, Armonk, NY, USA) was applied for statistical analysis. GraphPad Prism 8 software was applied to draw Kaplan-Meier curves. A two-sided *P* < 0.05 was considered statistically significant.

## Results

### Patient Characteristics

Between January 1990 and December 2016, a series of 159 resected stage I–IIIA LELC cases were evaluated. The general characteristics are summarized in Table 1. For clinical features, the median age of the entire cohort was 55 years old (range: 27–75 years old). Males and females were at a comparable proportion (45.3 vs. 54.7%). Non-smoker (73.6%) accounted for most of the cases. The median value of preoperative albumin level was 42.7 g/L (range: 28.6–53.3 g/L). Most patients had normal preoperative albumin levels (88.7%) and CEA level (95%). Almost all the patients were diagnosed as poorly differentiated LELC (96.2%). Most patients had ELNs > 34 (83.0%). For IHC characteristics, there were higher expression levels of CK (95.9%), CK5/6 (99.3%), P63 (97.1%), and EBER (99.3%), and lower expression levels of CK7 (96.7%) and TTF-1 (95.1%). Most cases were EGFR-wild (97.0%) and ALK-wild (97.8%).

**Table 1:** Clinicopathological characteristics of included LELC patient.

**Clinical characteristic**	**No. patients (%)**
**Age**	
Median (range)	55 (27-77)
≤ 60	115 (72.3)
>60	44 (27.7)
**Sex**	
Male	72 (45.3)
Female	87 (54.7)
**Smoking**	
Non-smoker	117 (73.6)
Smoker	42 (26.4)
**Tumor history**	
No	135 (84.9)
Yes	24 (15.1)
**Preoperative albumin level (g/L)**	
Median (range)	42.7 (28.6–53.3)
<35	18 (11.3)
≥35	141 (88.7)
**CEA (μg/ml)**	
<5	151 (95.0)
≥5	8 (5.0)
**Location**	
Central	42 (26.4)
Peripheral	117 (73.6)
**Morphology**	
Regular	35 (22.0)
Irregular	124 (78.0)
**Site**	
RUL	18 (11.3)
RML	41 (25.8)
RLL	28 (17.6)
LUL	21 (13.2)
LLL	51 (32.1)
**Surgical type**	
Lobectomy	125 (78.6)
Wedge resection	8 (5.0)
Bilobectomy	10 (16.3)
Pneumonectomy	16 (10.1)
**Diameter**	
Median (range)	4.0 (0.6–11.0)
**Grade**	
Well differentiation	0 (0.0)
Moderately differentiation	0 (0.0)
Poor differentiation	153 (96.2)
Undifferentiation	6 (3.8)
**Examined lymph nodes**	
Median (range)	22 (1–73)
≤ 34	27 (17.0)
>34	132 (83.0)
**Positive lymph nodes**	
Median (range)	1 (0–16)
≤ 4	137 (86.2)
>4	22 (13.8)
**T stage**	
1	45 (28.3)
2	75 (47.2)
3	28 (17.6)
4	11 (6.9)
**N stage**	
0	75 (47.2)
1	31 (19.5)
2	53 (33.3)
**TNM stage**	
I	52 (32.7)
II	41 (25.8)
III	66 (41.5)
**Adjuvant therapy**	
No	79 (49.7)
Yes ^a^	80 (50.3)
**CK (*****n*** **=** **73)**	
Positive	70 (95.9)
Negative	3 (4.1)
**CK 5/6 (*****n*** **=** **136)**	
Positive	135 (99.3)
Negative	1 (0.7)
**CK 7 (*****n*** **=** **91)**	
Positive	3 (3.3)
Negative	88 (96.7)
**TTF-1 (*****n*** **=** **103)**	
Positive	5 (4.9)
Negative	98 (95.1)
**P 63 (*****n*** **=** **139)**	
Positive	135 (97.1)
Negative	4 (2.9)
**EBER (*****n*** **=** **147)**	
Positive	146 (99.3)
Negative	1 (0.7)
**EGFR (*****n*** **=** **99)**	
Mutated	3 (3.0)
Wild	96 (97.0)
**ALK (*****n*** **=** **91)**	
Mutated	2 (2.2)
Wild	89 (97.8)

a *Adjuvant therapy includes chemotherapy (67 cases), radiotherapy (3 cases), chemoradiotherapy (8 cases), target therapy (1 case), and immunotherapy (1 case)*.

*LELC, lymphoepithelioma-like carcinoma; RUL, right upper lobe; RML, right middle lobe; RLL, right low lobe; LUL, left upper lobe; LLL, left low lobe; TTF-1, thyroid transcription factor-1; EBER, Epstein-Bar virus-encoded RNA; CK, creatine kinase; EGFR, epidermal growth factor receptor and ALK, anaplastic lymphoma kinase*.

A total of 2,757 ADC cases and 1,331 SCC cases from SYSUCC between January 2001 and December 2016 were also included. After PSM, there were 113 pairs in the LELC&ADC group and 91 pairs in the LELC&SCC group. The clinicopathological features of these tumors before and after PSM are listed in Supplementary Table S1 (ADC vs. LELC) and Supplementary Table S2 (SCC vs. LELC). After PSM, all covariates were well-balanced among these pairs.

### Cox Regression Analysis

Regarding OS, a univariate analysis revealed that age ≤60, preoperative albumin level ≥35, lobectomy surgical type, regular morphology, ELNs ≤34, PLNs ≤4, and N0 stage were favorable prognostic factors (Table 2). Multivariate analysis confirmed that age ≤60, preoperative albumin level ≥35, lobectomy surgical type, regular morphology, and N0 stage were independent predictors favoring OS (Table 2).

**Table 2:** Univariate and multivariate COX proportional hazard model analysis for overall survival.

**Characteristic**	**Univariate analysis**			**Multivariate analysis^a^**		
	**HR**	**95% CI**	***P***	**HR**	**95%CI**	***P***
Age			**0.048**			**0.014**
≤ 60	Ref			Ref		
>60	2.188	1.008–4.747		2.886	1.235–6.743	
Sex			0.212			
Male	Ref					
Female	1.648	0.753–3.606				
Smoking			0.597			
Non-smoker	Ref					
Smoker	1.250	0.547–2.856				
Tumor history			0.567			
No	Ref					
Yes	0.704	0.212–2.341				
Preoperative albumin level (g/L)			**<0.001**			**<0.001**
<35	Ref			Ref		
≥35	0.145	0.066–0.319		0.168	0.072–0.392	
CEA (μg/ml)			0.362			
<5	Ref					
≥5	0.980	0.612–1.952				
Location			0.374			
Central	Ref					
Peripheral	0.696	0.312–1.549				
Morphology			**0.005**			**0.006**
Regular	Ref			Ref		
Irregular	2.953	1.380–6.316		3.802	1.479–9.774	
Surgical type			**0.036**			**0.015**
Lobectomy	Ref			Ref		
Non-lobectomy	2.312	1.056–5.059		3.136	1.243–7.907	
Site			0.793			
RUL	Ref					
RML	1.067	0.215–5.299				
RLL	1.517	0.294–7.838				
LUL	2.187	0.424–11.291				
LLL	1.448	0.312–6.714				
Grade			0.422			
Poor differentiation	Ref					
Undifferentiation	0.045	0.002–85.872				
Examined lymph nodes			**0.040**			0.218
≤ 34	Ref			Ref		
>34	2.381	1.039–5.456		1.707	0.586–4.976	
Positive lymph nodes			**<0.001**			0.702
≤ 4	Ref			Ref		
>4	5.714	2.596–12.579		1.306	0.384–4.450	
T stage			0.054			
1	Ref					
2	1.171	0.433–3.167				
3	1.319	0.403–4.324				
4	4.482	1.361–14.760				
N stage			**0.001**			**0.021**
0	Ref			Ref		
1	2.041	0.548–7.604		2.139	0.524–8.728	
2	5.985	2.219–16.141		5.643	1.637–19.447	
Adjuvant therapy			0.597			
No	Ref					
Yes^b^	1.227	0.574–2.623				

*The meaning of bold values is two-sided P < 0.05*.

a *Variables with P < 0.05 were included in the multivariate analysis*.

b *Adjuvant therapy includes chemotherapy (67 cases), radiotherapy (3 cases), chemoradiotherapy (8 cases), target therapy (1 case), and immunotherapy (1 case)*.

*HR, hazard ratio; CI, confidence interval; RUL, right upper lobe; RML, right middle lobe; RLL, right low lobe; LUL, left upper lobe; LLL, left low lobe*.

Univariate analysis of DFS demonstrated that albumin level ≥35, did not perform adjuvant therapy, PLNs ≤4 and N0 stage had favorable impacts on DFS (Table 3). Multivariate analysis confirmed that albumin level ≥35, PLNs ≤4, and N0 stage were independent favorable prognostic factors (Table 3).

**Table 3:** Univariate and multivariate COX proportional hazard model analysis for disease-free survival.

**Characteristic**	**Univariate analysis**			**Multivariate analysis^a^**		
	**HR**	**95% CI**	***P***	**HR**	**95%CI**	***P***
Age			0.102			
≤ 60	Ref					
>60	1.673	0.904–3.097				
Sex			0.110			
Male	Ref					
Female	1.653	0.893–3.061				
Smoking			0.272			
Non-smoker	Ref					
Smoker	1.427	0.756–2.693				
Tumor history			0.450			
No	Ref					
Yes	0.698	0.275–1.773				
Preoperative albumin level (g/L)			** <0.001**			**0.008**
<35	Ref			Ref		
≥35	0.278	0.140–0.554		0.382	0.187–0.781	
CEA (μg/ml)			0.482			
<5	Ref					
≥5	0.861	0.775–1.414				
Location			0.650			
Central	Ref					
Peripheral	0.861	0.450–1.645				
Morphology			0.071			
Regular	Ref					
Irregular	1.796	0.951–3.390				
Surgical type			0.267			
Lobectomy	Ref					
Non-lobectomy	1.457	0.750–2.832				
Site			0.376			
RUL	Ref					
RML	2.160	0.478–9.754				
RLL	2.163	0.449–10.418				
LUL	4.071	0.879–18.849				
LLL	2.628	0.601–11.499				
Grade			0.310			
Poor differentiation	Ref					
Undifferentiation	0.046	0.005–17.781				
Examined lymph nodes			0.454			
≤ 34	Ref					
>34	1.324	0.636–2.755				
Positive lymph nodes			** <0.001**			**0.040**
≤ 4	Ref			Ref		
>4	4.431	2.339–8.392		2.202	1.035–4.685	
T stage			0.341			
1	Ref					
2	1.395	0.635–3.064				
3	1.791	0.727–4.409				
4	2.578	0.861–7.716				
N stage			** <0.001**			**0.026**
0	Ref			Ref		
1	2.329	0.898–6.040		2.150	0.819–5.639	
2	5.483	2.572–11.688		3.272	1.380–7.758	
Adjuvant therapy			**0.007**			0.063
No	Ref			Ref		
Yes^b^	2.393	1.268–4.517		1.853	0.968–3.546	

*The meaning of bold values is two-sided P < 0.05*.

a *Variables with P <0.05 were included in the multivariate analysis*.

b *Adjuvant therapy includes chemotherapy (67 cases), radiotherapy (3 cases), chemoradiotherapy (8 cases), target therapy (1 case), and immunotherapy (1 case)*.

*HR, hazard ratio; CI, confidence interval; RUL, right upper lobe; RML, right middle lobe; RLL, right low lobe; LUL, left upper lobe; LLL, left low lobe*.

### Nomogram

The nomogram for OS, formulated based on the statistically significant factors from the multivariate analysis, showed that N stage was the strongest predictor, followed by preoperative albumin level and tumor morphology (Supplementary Figure S1). The C-index of the nomogram was 0.86 [95% confidence interval (CI): 0.91–0.81]. The nomogram for DFS was also developed, and it revealed that N stage was also the strongest predictor, followed by preoperative albumin level and PLNs (Supplementary Figure S2). The C-index of the nomogram was 0.75 (95% CI: 0.68–0.82).

### Survival

**Figure 1:**
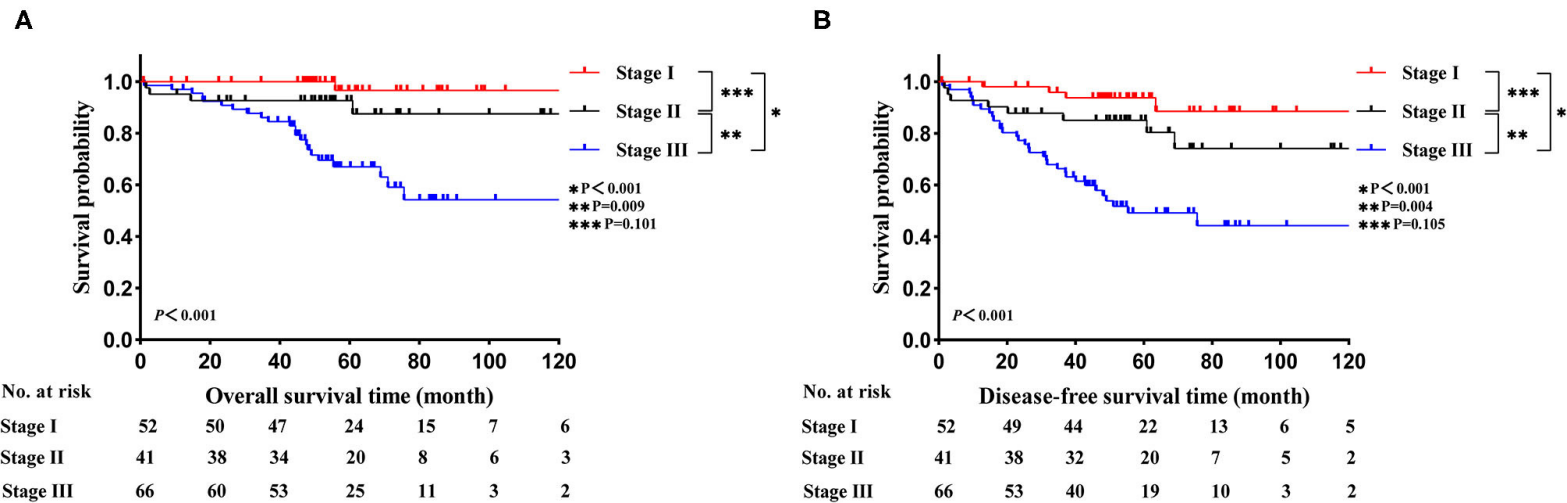
Kaplan-Meier estimates of survival in LELC across different TNM stages. **(A)** OS; **(B)** DFS. LELC, lymphoepithelioma-like carcinoma; TNM, tumor-node-metastasis; OS, overall survival; DFS, disease-free survival.

In the LELC cohort, the median follow-up time was 55.6 months (range: 0.9–209.9 months). The 3-, 5-, and 10-year OS rates were 92.1, 83.1, and 76.1%, respectively. The 3-, 5-, and 10-year DFS rates were 81.1, 72.7, and 66.1%, respectively. With regard to LELC patients, Kaplan-Meier curves of OS and DFS across different TNM stages are displayed in Figure 1 (Figure 1A: OS; Figure 1B: DFS). The OS and DFS seemed better in stage I group than stage II group, but the differences were not statistically significant (OS: *P* = 0.101; DFS: *P* = 0.105). Moreover, stage I and stage II cases enjoyed high levels of survival than stage III cases (all *P* < 0.05).

**Figure 2:**
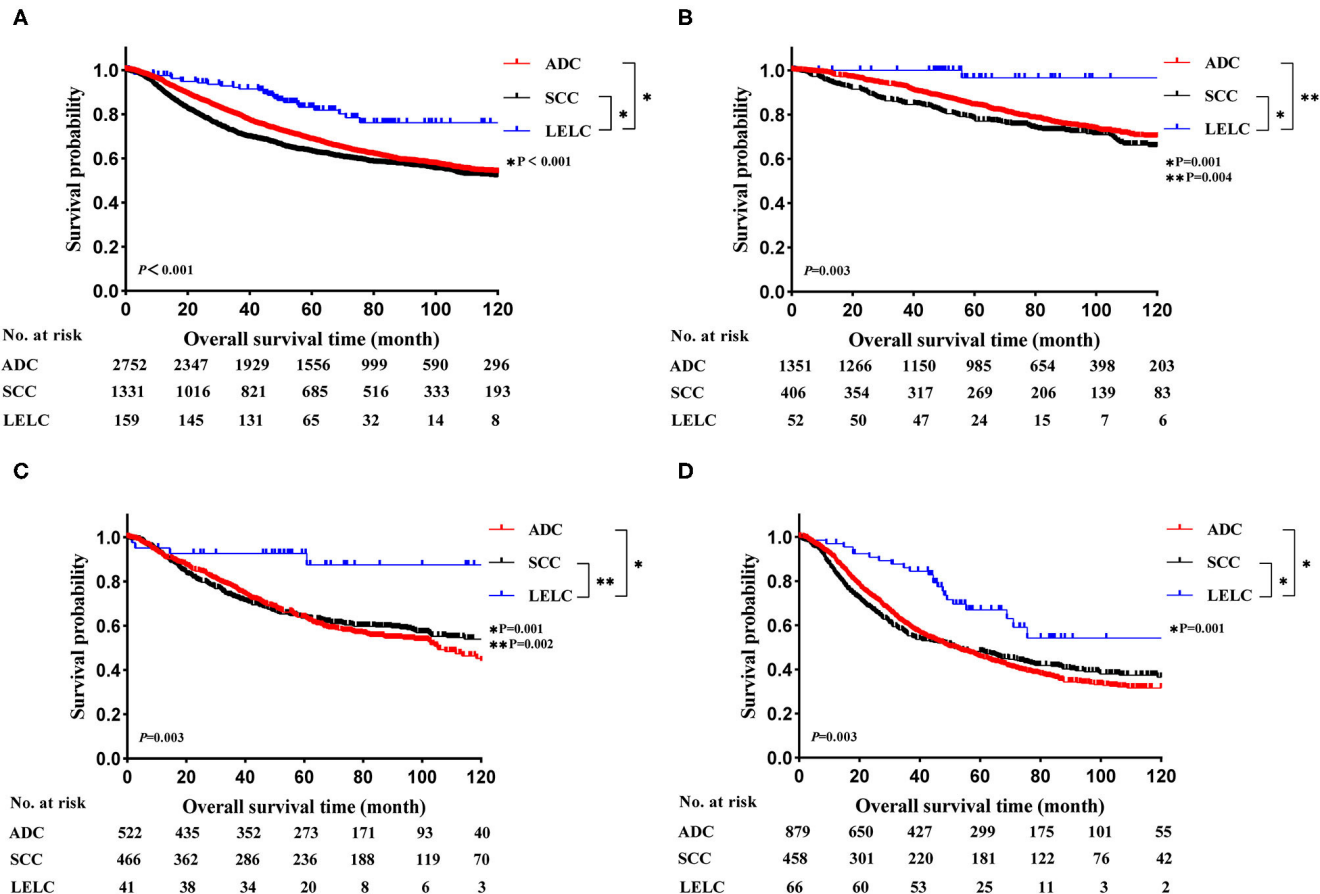
Kaplan-Meier estimates of OS in LELC vs. other NSCLCs across different TNM stages. **(A)** The entire cohort; **(B)** stage I cohort; **(C)** stage II cohort; **(D)** stage III cohort. LELC, lymphoepithelioma-like carcinoma; ADC, adenocarcinoma; SCC, squamous cell carcinoma; NSCLC, non-small cell lung cancer; OS, overall survival.

**Figure 3:**
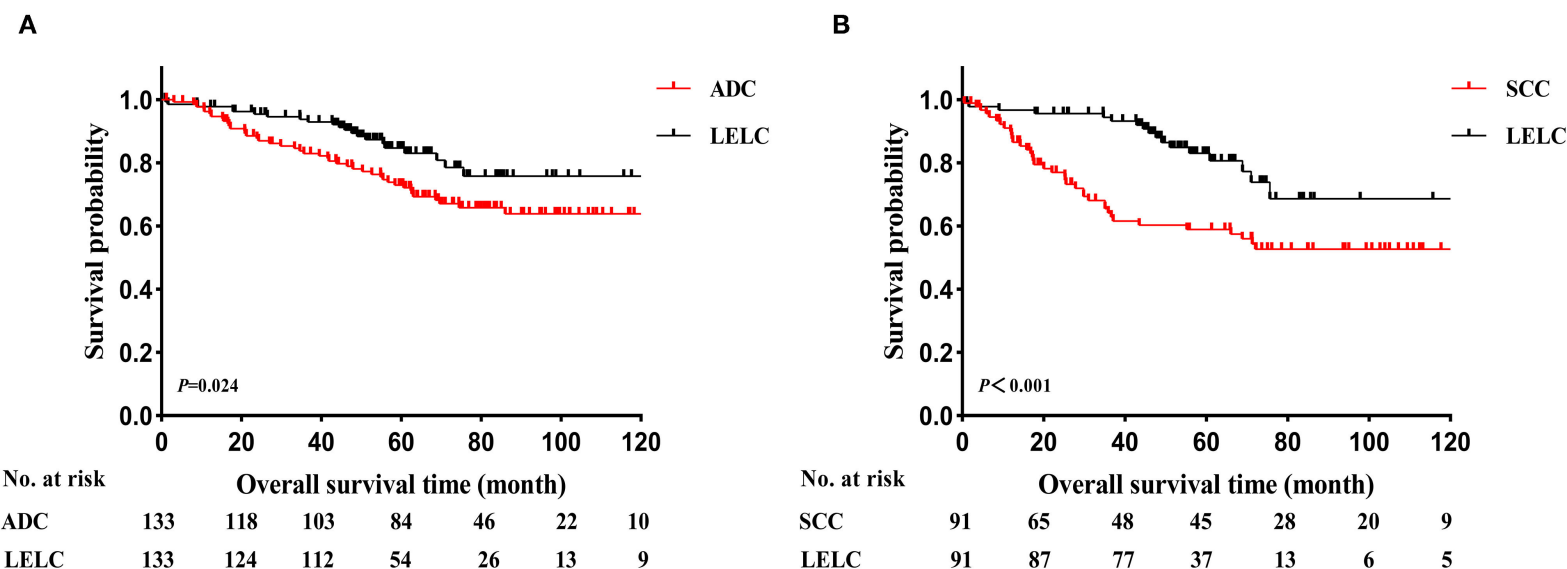
Kaplan-Meier estimates of OS in LELC vs. other NSCLCs after PSM. **(A)** LELC vs. ADC and **(B)** LELC vs. SCC. LELC, lymphoepithelioma-like carcinoma; ADC, adenocarcinoma; SCC, squamous cell carcinoma; NSCLC, non-small cell lung cancer; PSM, propensity score matching; OS, overall survival.

Before PSM, LELC had the best OS outcomes, followed by ADC and SCC (LELC vs. ADC, *P* < 0.001; LELC vs. SCC, *P* < 0.001; Figure 2A). In further analyses, significant differences were also found among LELC, ADC, and SCC in OS divided by TNM stages (Figure 2B: stage I, *P* = 0.003; Figure 2C: stage II, *P* = 0.003; Figure 2D: stage III, *P* = 0.003). After PSM, the 5-year OS rate of LELC was superior to those of ADC (84.7 vs. 73.0%; *P* = 0.024; Figure 3A) and SCC (83.0 vs. 58.9%; *P* < 0.001; Figure 3B).

## Discussion

In the present study, the patient characteristics, survival and prognosis of resected stage I–IIIA LELC were retrospectively investigated. Our data demonstrated that LELC was more prevalent in younger patients and non-smokers, with no obvious gender predisposition. In addition, LELC is a poorly differentiated disease that lacks typical driver gene mutations and is positive for IHC indicators of squamous cell lineage. In further analyses, LELC had a better survival outcome than other common lung cancers both before and after PSM. Finally, multivariate analyses revealed that both early N stage and preoperative albumin level ≥35 were prognostic factors favoring OS and DFS.

In previous study, several clinical series suggested that LELC is often identified in younger non-smokers (4, 12, 20), and there was no sexual predilection (4, 20, 21), which was akin to our findings (Supplementary Table S3). The abovementioned result suggested that unlike SCC, smoking might not be the main etiology of LELC (7, 12). Most tumors in our cohort were peripheral and had irregular morphology, echoing previous reports (22, 23), but conflicting with Qin et al.'s study (7). A large proportion of patients were diagnosed as locally advanced diseases (67.3%) which is akin to Zhou et al.'s study (6) and Qin et al.'s study (7). One of reasons postulated to account for this phenomenon was that patients diagnosed with LELC between 1990 and 2016, a period when lung cancer screening was still inadequate and people's awareness was still low in China, from our center were included in this study. This might interpret, to a certain extent, high proportion of advanced diseases.

In our study, almost all the cases were diagnosed as poorly differentiated disease, which was in accordance with previous findings that LELC is characterized by poorly differentiated tumor cells with prominent nucleoli and large vesicular nuclei (23, 24). IHC data showed that our results were similar to those of Jiang et al., where the authors investigated 43 resected LELC patients and concluded that the tumor is typically positive for CK, CK5/6, and P63, which suggests squamous cell lineage, but is negative for TTF-1 and CK7 (25) (Supplementary Table S3). Similar scenarios were also seen in Qin et al.'s study (7) and Liang et al.'s study (4) (Supplementary Table S3). Owing to the similar morphology and IHC indicators, LELC is often misdiagnosed as SCC (26). Previous reports demonstrated that the presence of EBV in the nuclei of LELC tumor cells is critical for diagnosis. This can be confirmed by EBER *in situ* hybridization testing (8, 27). In our research, EBER was positive in 99.3% of all the tested patients. From our perspective, if the patient originated from an area with a prevalence of EBV infection and presented with a peripheral lung mass, EBER testing was preferred in the pretreatment examination.

In our study, molecular testing revealed that LELC lacked target agent-sensitive mutations (EGFR and ALK). In the study by Hong et al., the authors explored the genetic landscape of LELC and demonstrated a low percentage of typical driver mutations, such as EGFR, BRAF, and KRAS (28). The same scenarios were also observed in Wang et al.'s study (29) and Chang et al.'s study (30). The results above indicated that typical driver gene mutations, the main etiology of other common NSCLCs, might not play a critical role in the carcinogenesis of LELC (31). Furthermore, EGFR or ALK-targeted agents might not be suitable in the neoadjuvant or adjuvant therapy of advanced LELC.

Our data demonstrated that both the OS and DFS of LELC showed a stepwise deterioration with the increase of TNM stage. From our perspectives, although the current TNM staging system is established based on the prognosis of common NSCLC subtypes, it is still efficient to predict survival of this rare disease. The nomogram also confirmed that the N stage was the strongest predictor. Before PSM, the OS of LELC was better than those of ADC and SCC across different TNM stages. After PSM, our study showed that the OS of LELC was also superior than those of ADC and SCC. Consistent with our results, He et al. assessed 62 LELC patients and suggested that LELC patients enjoy a higher level of survival when compared with ADC, SCC, and large cell lung cancer (22). However, their conclusions might be impaired by the relatively small cohort size. In line with our findings, Chen et al. also reviewed 42 LELCs and 132 SCCs and concluded that LELC patients present longer progression-free survival than SCC patients. Nevertheless, OS, the gold standard of evaluating the efficacy of treatment modality, was lacking in their research. In the study by Zhou et al. the authors compared the OS of LELC with ADC, SCC, and neuroendocrine tumors (6). Their data suggested that the OS of LELC is superior to those of SCC and neuroendocrine tumors but comparable to that of ADC (6), which was contradicted with ours. However, PSM method was not used in their research, which may confer bias. One plausible explanation for the results observed in our study is that compared with other common NSCLCs, LELC was dominant in younger and non-smoker patients. Smoking leads to more preoperative complications such as hypertension (32), coronary heart disease (33), and respiratory diseases (34), which might reduce life expectancy.

The multivariate analysis revealed that N stage and preoperative albumin level were correlated with both OS and DFS in our study. It is evidenced that nodal stage is an important influencing factor for LELC patient survival (8, 12, 15). For albumin level, Liang et al. investigated the outcomes of 52 resected LELCs and demonstrated that the serum albumin level was an independent prognostic factor (4), which was similar to our findings. Surprisingly, T stage and tumor grade, two important prognosis predictors in other NSCLCs, were not correlated with OS and DFS in our study, suggesting that the natural course and biology of LELC might be different from those of other common NSCLCs. The nomograms, developed based on the results of multivariate analyses of OS and DFS, showed good performances. The nomogram, a simple but effective statistical predictive tool with visual graphics, is able to integrate multiple predictive factors and decode the probability of an event more easily than ordinary evaluation methods. This is the first attempt to construct prediction nomograms based on clinicopathologic data of cases with LELC. Considering that all prognostic factors involved in our nomogram are easily obtained clinical data, it is convenient and practical for clinicians to perform a personal prediction of survival. With the help of this nomogram, we could define the LELC patients with an enhanced likelihood of poor survival. As is widely acknowledged that there are still many developing countries such as China in the world. Some patients who are in relatively poor economic situation and living in rural area far away may be suffered from LELC. The expensive follow-up examinations and long distances form major obstacles for these patients to get scheduled follow-up examinations. Based on our nomogram, we recommended that a closer surveillance or more intensive care might be essential for the high-risk population, and low-risk LELC patients may need less intensive surveillance which could not only reduce economic burden, but also reduce irradiation exposure.

To the best of our knowledge, this study represents the first comprehensive and concurrent analysis of resected stage I–IIIA LELC. In addition, the virtues of this study were that it included the largest cohort size and had a long-term follow-up. Additionally, the evaluation of a wide range of clinicopathological variables allowed us to better understand the demographic trends and prognosis of the disease.

However, our study also had some limitations. First, DFS comparations among LELC, ADC and SCC both before PSM and after PSM were lacking in our study due to the fact that our database does not contain any follow-up information about DFS of ADC and SCC. Second, in the era of precision therapy, molecular indicators such as PD-1, PD-L1, KRAS, and BRAF were not involved in our study. Third, despite the significant advantages provided by a larger case number than has ever been reported before, the cohort size was still limited and it was tough do more detailed analyses. Finally, the retrospective nature may have contributed to selection bias. Further efforts on prospective data collection and incorporation of the abovementioned factors are warranted.

## Conclusions

In conclusion, LELC is a rare distinct subtype of NSCLC that prevails in young non-smokers. It was also a poorly differentiated disease that lacked typical driver gene mutations and was positive for squamous cell lineage IHC indicators. Further analyses revealed that LELC had a better survival outcome than ADC and SCC.

## Data Availability Statement

The original contributions presented in the study are included in the article/Supplementary Material, further inquiries can be directed to the corresponding authors.

## Ethics Statement

The studies involving human participants were reviewed and approved by the Ethics Committee of Sun Yat-Sen University Cancer Center (SYSUCC). The patients/participants provided their written informed consent to participate in this study.

## Author Contributions

LL and X-MD: conception and design. LL: administrative support. W-TZ, M-XG, X-JJ, and X-LT: provision of study materials or patients. R-RJ and X-LF: collection and assembly of data. X-MD: data analysis and interpretation. All authors manuscript writing and final approval of manuscript.

## Conflict of Interest

The authors declare that the research was conducted in the absence of any commercial or financial relationships that could be construed as a potential conflict of interest.

## Publisher's Note

All claims expressed in this article are solely those of the authors and do not necessarily represent those of their affiliated organizations, or those of the publisher, the editors and the reviewers. Any product that may be evaluated in this article, or claim that may be made by its manufacturer, is not guaranteed or endorsed by the publisher.

## Supplementary Material

Supplementary Figure S1 Nomogram for predicting the 3-, 5-, and 10-year OS in LELC patients. LELC, lymphoepithelioma-like carcinoma; OS, overall survival.

Supplementary Figure S2 Nomogram for predicting the 3-, 5-, and 10-year DFS in LELC patients. LELC, lymphoepithelioma-like carcinoma; DFS, disease-free survival.

Supplementary Table S1 General clinical characteristics of ADC and LELC before and after PSM.

Supplementary Table S2 General clinical characteristics of SCC and LELC before and after PSM.

Supplementary Table S3 Demography of previous reported LELC cases.

## Abbreviations

LELC: lymphoepithelioma-like carcinoma
NSCLC: non-small cell lung cancer
WHO: World Health Organization
EBV: Epstein-Barr virus
OS: overall survival
DFS: disease-free survival
ADC: adenocarcinoma
SCC: squamous cell carcinoma
IHC: immunohistochemistry
PSM: propensity score matching
SYSUCC: Sun Yat-sen University Cancer Center
AJCC: American Joint Committee on Cancer
ELNs: examined lymph nodes
PLNs: positive lymph nodes
C-index: concordance index
RUL: right upper lobe
RML: right middle lobe
RLL: right low lobe
LUL: left upper lobe
LLL: left low lobe
TTF-1: thyroid transcription factor-1
EBER: Epstein-Bar virus-encoded RNA
EGFR: epidermal growth factor receptor
ALK: anaplastic lymphoma kinase
CI: confidence interval
HR: hazard ratio.
