# Supplementary figures and images for: A Rare Subtype of Non-small Cell Lung Cancer: Report of 159 Resected Pathological Stage I–IIIA Pulmonary Lymphoepithelioma-Like Carcinoma Cases
Source: Front Surg. 2021 Oct 27;8:757085. doi: 10.3389/fsurg.2021.757085 (PMC8580194; doi:10.3389/fsurg.2021.757085)

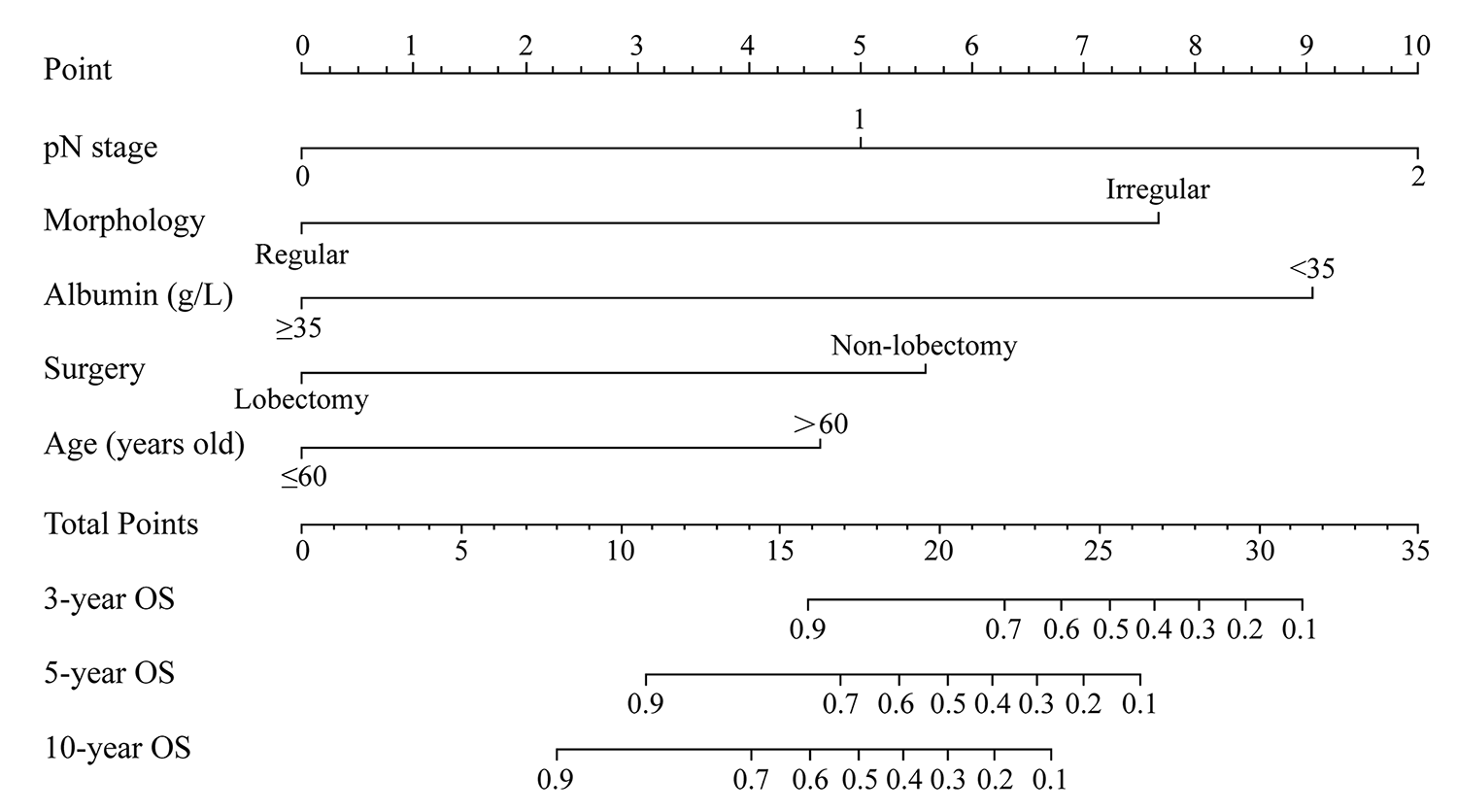

Supplement: Supplementary Figure S1 — Nomogram for predicting the 3-, 5-, and 10-year OS in LELC patients. LELC, lymphoepithelioma-like carcinoma; OS, overall survival. [file Image_1.TIF]

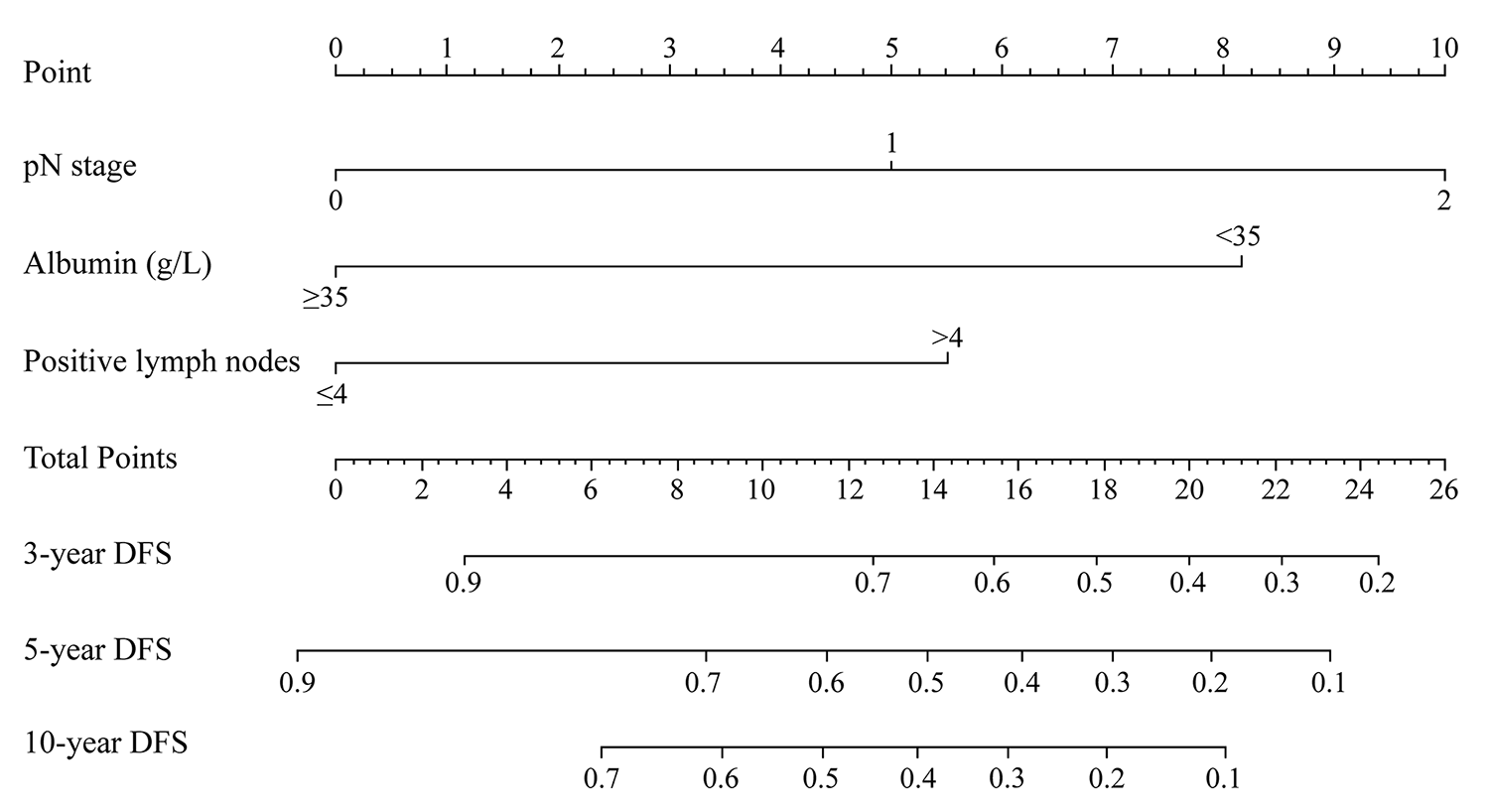

Supplement: Supplementary Figure S2 — Nomogram for predicting the 3-, 5-, and 10-year DFS in LELC patients. LELC, lymphoepithelioma-like carcinoma; DFS, disease-free survival. [file Image_2.TIF]
